# Fabrication and Optimization of Poly(ε-caprolactone) Microspheres Loaded with S-Nitroso-N-Acetylpenicillamine for Nitric Oxide Delivery

**DOI:** 10.3390/biomedicines12061363

**Published:** 2024-06-19

**Authors:** Syed Baseeruddin Alvi, Nooruddin Pracha, Mahmoud Shalaan, Pankaj Singh Dholaniya, Muhamad Mergaye, Divya Sridharan, Mahmood Khan

**Affiliations:** 1 Division of Basic and Translational Research, Department of Emergency Medicine, The Ohio State University, Columbus, OH 43210, USA; 2 Department of Biotechnology and Bioinformatics, School of Life Sciences, University of Hyderabad, Hyderabad 500046, India; 3 Department of Physiology and Cell Biology, The Ohio State University, Columbus, OH 43210, USA; 4 Davis Heart and Lung Research Institute, The Ohio State University, Columbus, OH 43210, USA

**Keywords:** microspheres, nitric oxide, sustained release, cardiomyocyte, anoxia

## Abstract

Heart disease is one of the leading causes of death in the United States and throughout the world. While there are different techniques for reducing or preventing the impact of heart disease, nitric oxide (NO) is administered as nitroglycerin for reversing angina or chest pain. Unfortunately, due to its gaseous and short-lived half-life, NO can be difficult to study or even administer. Therefore, controlled delivery of NO is desirable for therapeutic use. In the current study, the goal was to fabricate NO-releasing microspheres (MSs) using a donor molecule, S-Nitroso-N-Acetyl penicillamine, (SNAP), and encapsulating it in poly(ε-caprolactone) (PCL) using a single-emulsion technique that can provide sustained delivery of NO to cells over time without posing any toxicity risks. Optimization of the fabrication process was performed by varying the duration of homogenization (5, 10, and 20 min) and its effect on entrapment efficiency and size. The optimized SNAP-MS had an entrapment efficiency of ˃50%. Furthermore, we developed a modified method for NO detection by using NO microsensors to detect the NO release from SNAP-MSs in real time, showing sustained release behavior. The fabricated SNAP-MSs were tested for biocompatibility with HUVECs (human umbilical vein endothelial cells), which were found to be biocompatible. Lastly, we tested the effect of controlled NO delivery to human induced pluripotent stem-derived cardiomyocytes (hiPSC-CMs) via SNAP-MSs, which showed a significant improvement in the electrophysiological parameters and alleviated anoxic stress.

## 1. Introduction

Nitric oxide (NO) is a gaseous molecule endogenous to the human body with antimicrobial, smooth muscle-relaxing, and vasodilative properties [1]. In a physiological context, NO is also known for its angiogenic abilities as it causes capillary-like growths of endothelial cells [2]. NO has shown its biological capabilities specifically in wound healing and potential in treating cardiovascular implications [3]. In clinical settings, NO is commonly administered through nitroglycerin during emergent cases or by being inhaled [4,5]. Mechanistically, NO works by causing vasodilation via the NO/cyclic guanosine monophosphate (cGMP) signaling pathway [6]. In the human body, the NO molecule is initially formed from L-arginine from neuronal and endothelial cells via nitric oxide synthase, which is activated by calcium as well as calmodulin. The result of this enzymatic reaction forms NO, which then diffuses through the cell membrane of nearby cells. NO then proceeds to bind and activate guanylyl cyclase, forming the complex NO-guanylyl cyclase, which works to increase the synthesis of cGMP [7]. High levels of cGMP then proceed to have downstream effects on various effector molecules throughout the body including the cardiovascular system by promoting vasodilatory effects [8]. Aside from the various cardiovascular disease-related applications of NO, its use has also been extended to slowing the progress of neurodegenerative disease [9] and reducing post-surgical-related pulmonary hypertension [10], and it has also shown potential in slowing tumor growth, migration, and metastases in cancers [11]. Although these treatment modalities may be effective, and they have been known for a while, long-term or targeted NO administration is hindered due to its relatively short lifetime (a few seconds) and diffusional distance [12]. Additionally, excessive amounts of NO can be toxic and, in some cases, it can even promote tumors or malignant growth [13]. Recently, various donors have been developed and applied, which have made the release and application of NO more effective [12]. Glyceryl trinitrate (GTN) has been commonly used to treat angina pectoris [14]. However, a common problem that arises is the uncontrolled and untargeted release mechanism of NO, which gets distributed throughout the entire body. This untargeted release also exacerbates the possibility of toxicity within the body. For example, a common direct donor used in the treatment of hypertension and heart failure is Sodium Nitroprusside [15]. This drug is limited to only being administered parenterally due to the possibility of causing thiocyanate toxicity [16]. Other molecules such as mononitrate, isoamyl nitrite, and isosorbide dinitrate are all donors that have been used to release NO [17]. However, like GTN, all the aforementioned NO donors have drawbacks such as toxicity, clinical limitations due to temperature requirements, and the hinderance of enzyme interactions that would make NO use more harmful than effective [17]. Several NO donor molecules also provide an initial burst of NO that can be carcinogenic and not entirely practical [12]. As a result, a delivery modality is warranted that avoids these issues. One of the major concerns is effectively administering NO without posing significant toxicity as higher doses or excess amounts of NO have been shown to have detrimental effects and may lead to inflammation [18]. Thus, the controlled use and optimization of NO in the clinical setting hold the key to unlocking a new paradigm in the field of medicine.

**Figure 1:**
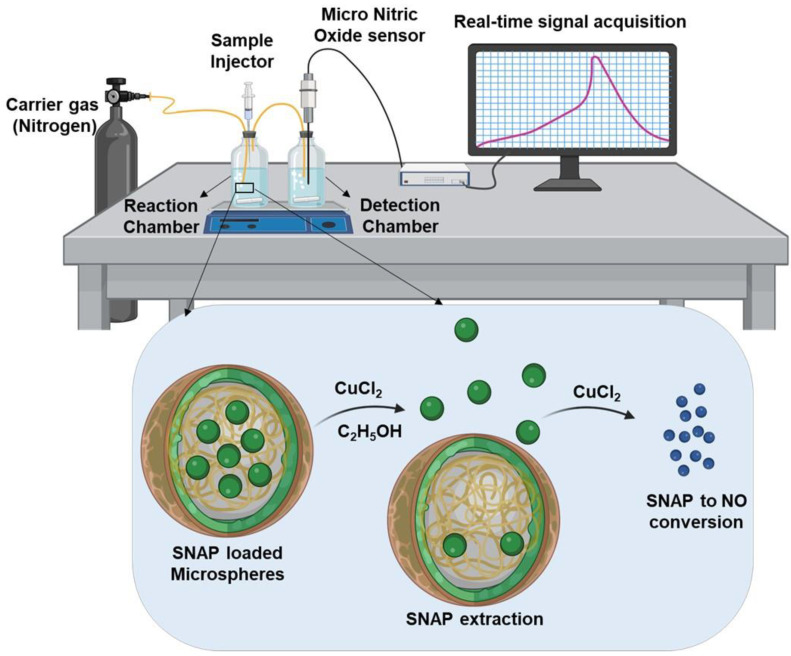
Schematic demonstrating the modified NO detection setup and the process of SNAP extraction and its conversion to NO for subsequent detection using carbon fiber NO sensors. (The figure was made using BioRender.com).

The myocardium requires specific attention for possible therapeutic strategies while also maintaining low levels of toxicity to avoid exacerbation of cardiovascular disease. The use of slow-release microspheres can help solve many of the problems as they allow for the gradual release of a NO donor molecule over time. This helps to decrease toxicity and allows for sustained benefits such as vasodilation and angiogenesis over time. In our study, we found the donor molecule, S-Nitroso-N-acetyl penicillamine (SNAP), to be an effective solution as it does not pose toxicity risks at low levels, addressing the current common drawback to NO delivery methods [19]. SNAP has shown many benefits including maintenance of blood pressure in animal models and helping regulate the immune response in shock [20,21]. The other limitation of using SNAP as a stand-alone delivery for NO is its poor stability in aqueous conditions; it is reported that SNAP can undergo rapid degradation (~24 h) in aqueous conditions [22]. Therefore, encapsulating SNAP in a polymer matrix can potentially improve the stability of SNAP by preventing its premature exposure to the aqueous environment. In terms of microsphere (MS) materials, a prior study has fabricated NO-releasing MSs using the polymer poly(lactic-co-glycolic acid) PLGA [12]. However, our study was focused on using poly(ε-caprolactone) (PCL) a potentially favorable polymer for MS fabrication [23]. When compared to PLGA, the PCL tends to have higher entrapment efficiency and can pose less of a toxicity risk, as PLGA degrades to lactic acid causing tissue inflammation [24,25]. Additionally, many studies have performed colorimetric or fluorometric detection of NO, which extensively relies on intermediates and may not be applicable in real-time detection. Therefore, our study developed a method to detect NO in real time by employing carbon fiber-based NO microsensors for higher sensitivity as shown in Figure 1.

## 2. Materials and Methods

### 2.1. Materials and Instrumentation

S-Nitroso-N-Acetyl-D L-Penicillamine (SNAP) was procured from Cayman Chemical (Ann Arbor, MI, USA). Pluronic F-127, Polycaprolactone (PCL) [Mw = 70,000], and Polyvinyl alcohol (PVA) [87–90% hydrolyzed, average molecular weight ~30–70 k] were obtained from Sigma Aldrich (Saint Louis, MO, USA). An Apollo Free Radical detector for Nitric Oxide (World Precision Instruments, Sarasota, FL, USA) was used. PowerLab, equipped with LabChart 5, was used for real-time signal acquisition (AD Instruments, Dunedin, New Zealand).

### 2.2. Fabrication of SNAP-Loaded Microspheres

The SNAP-loaded PCL-MSs were fabricated by following a single-emulsion method (W/O) as reported elsewhere. Briefly, 30 mg of PCL was dissolved in 1 mL of chloroform using a magnetic stirrer. Then, 10 mg Pluronic and SNAP were added to the same, forming an oil phase. The resulting mixture was added dropwise to a 25 mL aqueous phase (1% PVA). The solution was then homogenized at 20,000 RPM for 20 min to form an emulsion and later stirred for 24 h to evaporate the chloroform. The following day, the mixture was centrifuged at 1000 RPM, and the supernatant was collected for 3 rounds to remove the unloaded SNAP. Empty MSs were fabricated similarly as above but without the SNAP [23].

### 2.3. Characterization

#### 2.3.1. Size Optimization of SNAP-MSs

The size of SNAP-MSs was optimized by varying the homogenization time: the emulsion was homogenized for 5, 10, and 20 min and the samples were stirred for 24 h using a magnetic stirrer (100 rpm). The following day, an aliquot from each sample was dropped on a glass slide and imaged using a light microscope. Images were captured and the size of the microspheres was calculated using Image J software (version 1.54d). Dynamic light scattering (DLS) analysis was performed to confirm the size of the blank and SNAP-loaded microspheres, when subjected to 20 min homogenization. Later, the optimized sample was verified using scanning electron microscopy (SEM) to confirm sample size [26]. 

#### 2.3.2. Entrapment Efficiency and UV-Vis Spectroscopy

The entrapment efficiency of SNAP-MS was evaluated using a UV-Vis spectrometer (Beckman Coulter, DU 640B, Brea, CA, USA). The fabricated SNAP-MSs were centrifuged, and the so-formed pellet was dissolved in chloroform to extract the loaded SNAP and the absorbance was measured at 340 nm using quartz cuvettes for qantification. The baseline was normalized by using an equal amount of empty MSs, pelleted and dissolved in chloroform. The extracted SNAP was compared with the total amount of SNAP added and the respective percentage of entrapment was calculated as reported elsewhere [27]. The SNAP-loaded MSs, free SNAP dissolved in DMSO, and water were scanned using a plate reader (Synergy H1). 

#### 2.3.3. Effect of Homogenization on SNAP Stability

The free SNAP was subjected to high-speed homogenization and its stability was evaluated using a World Precision Instruments Apollo machine equipped with NO probes. At first, the standard curve of SNAP was made by following the manufacturer’s protocol: three doses of SNAP, i.e., 5.6, 11.2, and 22.4 μg, were spiked and the respective NO signals were recorded. Later, a freshly prepared SNAP solution was subjected to high-speed homogenization at 20,000 RPM for 20 min and the homogenized samples were then spiked to detect NO and compared with the non-homogenized sample to determine the degradation [28].

#### 2.3.4. Experimental Setup for Real-Time NO Measurement

A modified experimental setup was used to detect NO from SNAP-MSs as shown in Figure 1. The setup consisted of two separate chambers, i.e., reaction chamber and detection chamber. The reaction chamber was loaded with a solution of 0.1 M CuCl_2_ and 30% ethanol (to prevent foaming) in distilled water (to catalyze the breakdown of SNAP into NO). The reaction chamber was connected with an inlet tube, which was submerged into the solution, and an outlet tube above the surface (head space). The outlet tubing from the reaction chamber was then connected to the detection chamber containing distilled water and a NO microprobe (ISO NO, World Precision Instruments, USA). All the tube attachments were secured and connected to the nitrogen cylinder. Both the reaction and the detection chamber were placed on a magnetic stirrer and stirred at 50 RPM. The NO microprobe was connected to PowerLab (AD Instruments) and the signal was recorded using LabChart 5. A standard curve of NO was constructed using known concentrations of SNAP and spiking them into the reaction chamber with a continuous flow of nitrogen. Once the standard curve was established, the procedure was repeated by spiking SNAP-MSs to confirm the encapsulation and release of NO from the microspheres [29,30]. 

#### 2.3.5. Measurement of NO Released from SNAP-MSs

To evaluate NO release from the SNAP-MSs, the freshly prepared MSs were suspended in 1 mL distilled water and incubated at 37 °C. At each stipulated time point, 100 μL of the sample was drawn and tested for NO using the method described in the previous section. The gradual decline in the signal intensity on days 5 and 7 was compared with day 1 to estimate the % of SNAP (NO) remaining in the microspheres. Later, the same procedure was repeated to evaluate the temperature-triggered accelerated release of NO by incubating the samples at 60 °C for 24 h. The NO signal was recorded before and after the incubation time to compare the decline [31].

#### 2.3.6. Biocompatibility of SNAP-MS

The biocompatibility of SNAP-MS was tested using a human umbilical vein endothelial cell (HUVEC) cell line. The SNAP-MSs were treated at 100 μg/mL, and cell viability after 48 h was evaluated using live/dead fluorescence staining, and the percentage of viable cells (green) was quantified by Image J software [32]. 

#### 2.3.7. Effect of SNAP-MSs on hiPSC-CMs during Anoxic Stress

Multi-electrode array (MEA) analysis was performed as published previously [33] to assess the effect of SNAP-MSs on human induced pluripotent stem cell-derived cardiomyocytes (hiPSC-CMs). The cells were procured from FUJIFILM (cellular dynamics, Madison, WI, USA) and were cultured as per the company’s protocol. On day 7 of the culture, cells were trypsinized (0.25% Trypsin EDTA) and plated on a 24-well MEA plate (AXION biosystems), and the cells were allowed to stabilize for 48 h. Insert wells (8 μm) were modified to be placed inside the MEA wells that served as compartments for loading microspheres. Following the placement of inserts in the MEA wells, the test samples were loaded in the respective inserts (blank MS (250 μg/mL), free SNAP (125 μg/mL), SNAP-MS-L (250 μg/mL), and SNAP-MS-H (500 μg/mL)). Following the treatment, the cells were monitored for 48 h and later switched to a 5% CO_2_/95% N_2_ gas line to induce anoxia. The cells were subjected to anoxia for 48 h, before switching back to normal oxygenation (reoxygenation). The electrophysiological parameters like beat period, conduction velocity, and spike amplitude were recorded every 24 h and the change in each treatment condition was normalized to the baseline and reported as fold-change.

### 2.4. Statistical Analysis

Significant differences were determined using Kolmogorov–Smirnov test or one-way ANOVA followed by a post-hoc Dunnett’s test for comparison with the control. The data were considered statistically significant at *p* < 0.05.

## 3. Results and Discussion

**Figure 2:**
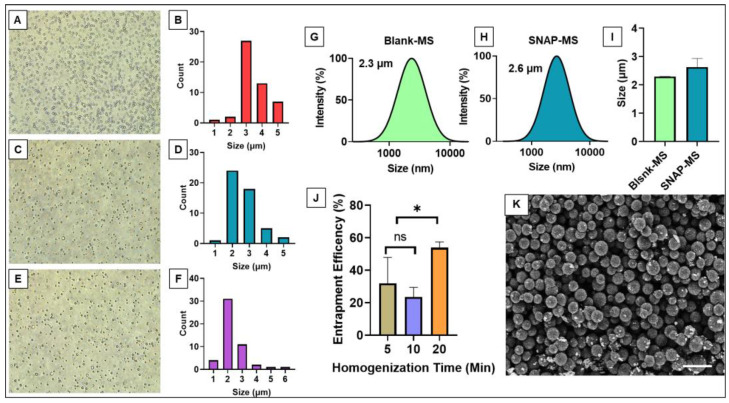
Characterization and optimization of SNAP-MSs. (**A**–**F**) Representative microscopic images of SNAP-MSs prepared with different homogenization durations (**A**,**B**) 5 min, (**C**,**D**) 10 min, and (**E**,**F**) 20 min, and respective count; image capture at 4X magnification. (**G**,**H**) Representative size distribution of blank MSs and SNAP MSs assessed by DLS. (**I**) Average size distribution of blank and SNAP-MS (*n* = 3); data is represented as mean ± SD. (**J**) Entrapment efficiency of SNAP-MS prepared with different homogenization time, (* *p* < 0.05, KS-Test). (**K**) Representative SEM image of optimized SNAP-MS formulation, scale bar 10 μm.

Microspheres (MSs) are broadly defined as microparticles ranging in size from 1–1000 μm [34]. Although fabricating MSs that fit within such a large size range may be relatively easy, our study sought to create MSs that were not only uniform in size but also within the range of 2–3 μm. We specifically sought this size range to give the MSs the potential ability to be administered into physiological systems, including some of the smallest capillaries in the human body that are around 5 μm [35]. Along with potentially administering the MSs, having them in a small uniform size can allow for conjugating them on different scaffold materials for implantation. In the current study, we have adopted a high-speed homogenization method for the fabrication of PCL-MSs by following an oil-in-water emulsion technique for loading SNAP into the PCL matrix (SNAP-MS). This fabrication technique allowed us to control the size of the MSs by modulating the time and speed of homogenization. The microscopic image analysis of the fabricated MSs indicated that 20 min of homogenization led to the most uniform distribution of MSs with a mean size of 2.26 μm. The 10-min and 5-min homogenization samples had a mean size of 2.57 μm and 3.38 μm, respectively. Although these size ranges would still be smaller than the smallest vessel in the human vasculature, as indicated in Figure 2A–F, the size distribution was not as uniform and stretched further out into the 5 μm size, which could potentially be too large for future applications. These results were consistent with earlier reports demonstrating the effect of homogenization on microsphere size [36]. We further evaluated the hydrodynamic diameter of the blank MS (2.32 ± 0.02 μm) and SNAP- MS (2.6 ± 0.3 μm) using DLS and, as shown in Figure 2G–I, the results were in alignment with the earlier report.

We also found that the entrapment efficiency was significantly improved (~52.38%) when the emulsion was homogenized for 20 min then shorter periods as shown in Figure 2J. This could be due to a longer homogenization duration allowing complete mixing of SNAPs with the PCL matrix. When compared to other studies, our single-emulsion technique was significantly better than PLGA-based MSs using DETA NONOate as a NO donor where the encapsulation efficiency was poor [37]. Lastly, the optimized sample of SNAP-MS was imaged using SEM to confirm the morphology of the fabricated MSs; as shown in Figure 2K, the microspheres were uniform and spherical. Our results demonstrate that using PCL-MSs for encapsulating SNAP is an efficient and superior delivery system than earlier reported studies.

**Figure 3:**
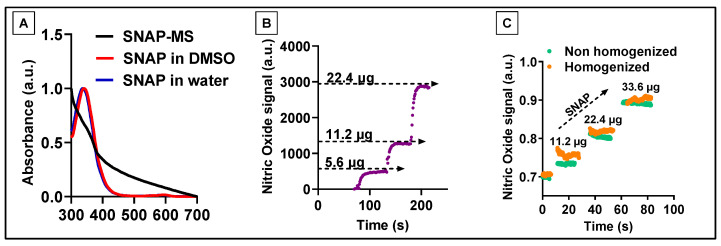
Characterization and stability assessment of SNAP. (**A**) UV-Vis spectroscopy of SNAP-MS and free SNAP dissolved in DMSO and water, (**B**) Standard dose–response of SNAP dissolved in water using the conventional NO detection method. (**C**) Stability of SNAP before and after high-speed homogenization.

The fabricated SNAP-MSs were then subjected to UV-Vis spectroscopy to confirm the loading, as shown in Figure 3A. SNAP dissolved either in DMSO or water showed a characteristic absorbance peak at ~340 nm, whereas after encapsulation into PCL-MS, a wide hump was noted around 340–400 nm confirming the presence of SNAP in the polymer matrix. However, to confirm whether the encapsulated SNAP was stable and did not undergo any degradation during the fabrication, it was essential to evaluate the stability of SNAP. Many NO donor molecules like nitroglycerine are reported to be labile and are known to be influenced by environmental conditions [38]. We sought to evaluate the stability of SNAP by subjecting it to formulation conditions like high-speed homogenization. This was performed by following the conventional NO detection method using NO microprobes [39], as shown in Figure 3B. Firstly, a standard curve was constructed using free SNAP by introducing incremental doses of SNAP. There was a gradual increase in the NO signal with each subsequent addition of SNAP. Later, a stock solution of SNAP was divided into two, and one of the samples was subjected to high-speed homogenization at 20,000 RPM for 20 min while the non-homogenized sample was placed at 4 °C for the same duration. After homogenization, both the samples were tested by creating similar standard curves. As shown in Figure 3C, the magnitude of NO signals generated were similar in both the samples. These results confirmed that SNAP when subjected to fabrication conditions like high-speed homogenization did not undergo rapid degradation. After confirming the stability, we sought to evaluate the release of NO from SNAP-MS by following the same conventional method. When the SNAP-MSs were introduced to the chamber containing the NO microprobe, it produced a strong signal that plateaued instantly and showed no decline even after 24 h of continuous monitoring (Figure S1). This suggested that the SNAP-MSs altered the sensitivity of microsensors and the signal generated was an artifact; therefore, we sought to modify the method of NO detection by separating the sensor from the sample to avoid any interference and directly detecting NO gas in real time (Figure 1). Our modified setup consisted of reaction chambers and a detection chamber; these chambers were connected to a continuous nitrogen gas line. The reaction chamber allowed the extraction of SNAP from SNAP-MSs and its conversion to NO gas. The generated NO gas was then carried to the detection chamber via the N_2_ line for real-time electrochemical detection of NO by microsensors. This allowed for NO gas to interact with the sensor without being affected by microsphere excipients.

**Figure 4:**
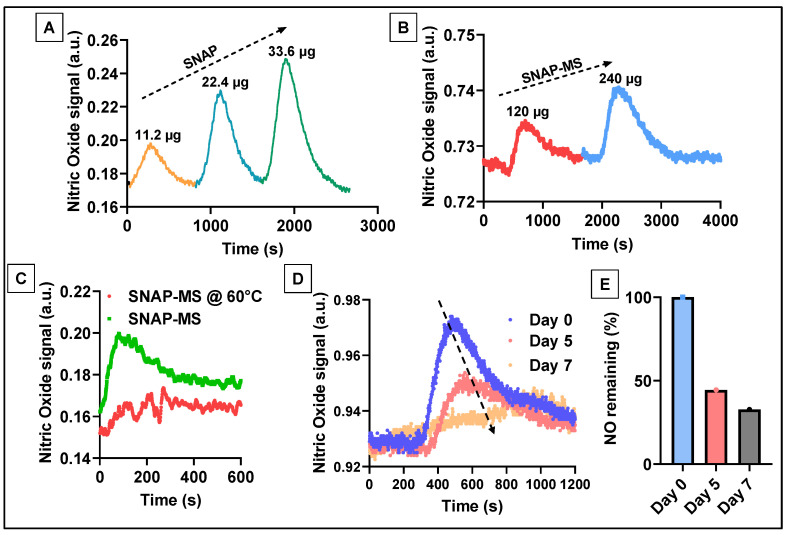
Validation of NO microsensor using the modified method and sustained release from SNAP-MSs. (**A**) Standard dose–response of SNAP using the modified NO detection method. (**B**) Dose response of SNAP-MSs. (**C**) Accelerated NO release from SNAP-MSs at 60 °C. (**D**,**E**) Detection of NO released from SNAP-MSs at different time points and quantification based on the AUC.

**Figure 5:**
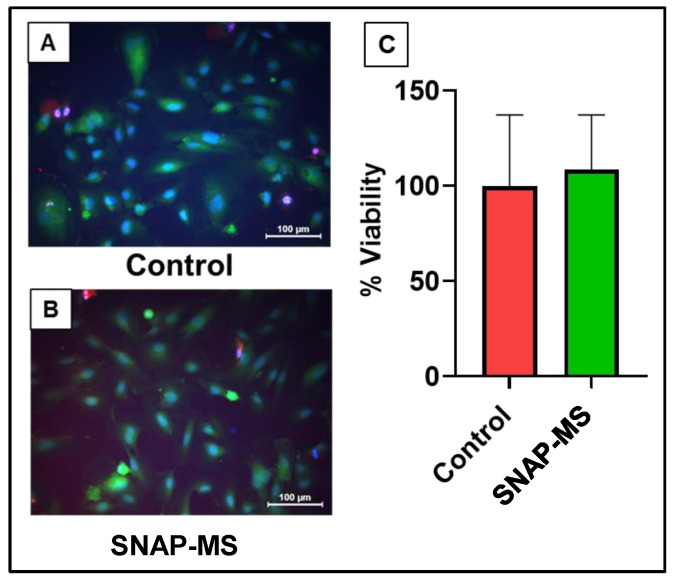
Biocompatibility of SNAP-MSs on HUVEC cell line. (**A**,**B**) Representative fluorescence microscopy of HUVEC cells treated with SNAP-MSs for live/dead staining (green/red, respectively). (**C**) Quantification of cell viability based on the green fluorescence.

We first validated the response of free SNAP in the modified setup. As shown in Figure 4A, the NO signal generated showed both an upward and a downward trend demonstrating the total duration of NO being in contact with the sensor at each dose. The peak magnitude was also linear with each tested dose. These findings validated the responsiveness of the NO microprobe in the modified setup. Later, the SNAP-MSs were tested in the same way and Figure 4B shows the NO signal generated in response to each dose. This confirmed that by using the modified method NO signal from the MSs could be detected without any interference. Later, we performed an accelerated release of NO by exposing the SNAP-MSs to elevated temperature. As the melting temperature of PCL is about 60 °C, maintaining the SNAP-MSs at this temperature leads to accelerated release of SNAP. We observed a significant decline in the NO signal post-24 h incubation when compared to the control, confirming the temperature-triggered release of NO from SNAP-MSs (Figure 4C) [40]. Lastly, we performed a sustained release experiment based on the earlier validation by incubating freshly prepared SNAP-MSs at 37 °C for 7 days, and at days 1, 5, and 7, 300 μL of the sample was drawn and tested for NO signal. As shown in Figure 4D, a significant and gradual decline was observed with longer incubation time points, suggesting a sustained release of NO from the SNAP-MSs was occurring. The area under the curve was calculated and compared with the day 0 sample to estimate the % of SNAP remaining by the end of day 7 (Figure 4E). These results demonstrate that the SNAP-MS could exhibit a sustained release behavior with about ~50% of NO release in five days. Our results demonstrate that by encapsulating SNAP in PCL microspheres better control over NO release can be achieved when compared to biopolymers [31]. Our results are in line with earlier published literature findings using a chemiluminescence nitric oxide analyzer, where an initial burst release of NO was observed followed by sustained release behavior when SNAP was used as a donor molecule [41]. Lastly, to test the biocompatibility of SNAP-MS, a live/dead cell assay was performed on HUVECs over 48 h, which indicated nearly identical levels of live cells in HUVECs treated with SNAP-MSs versus the control (Figure 5A–C). These results indicate that the SNAP-MSs and the amount of NO being released are biocompatible with endothelial cells.

**Figure 6:**
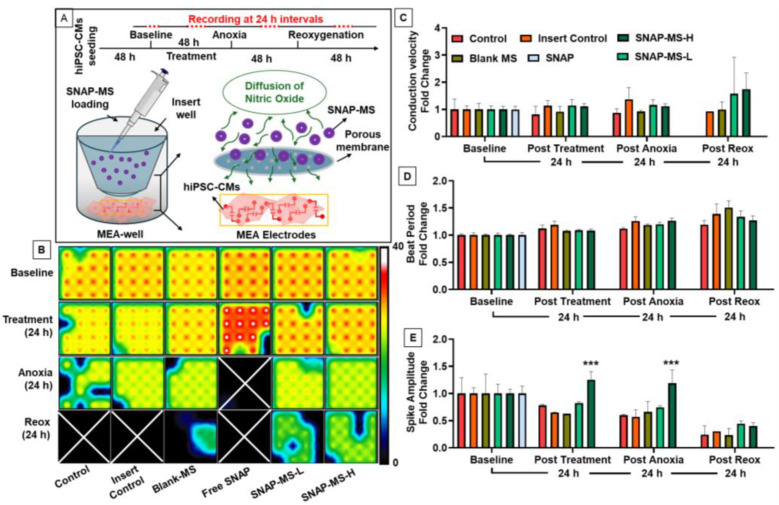
Protective effect of SNAP-MSs on hiPSC-CMs against anoxic stress. (**A**) Schematic representation of the experimental design. (**B**) Representative heat map of MEA well electrodes demonstrating the beat period, where the white cross represents loss of cell electrical activity. (**C**–**E**) Fold change in conduction velocity, beat period, and spike amplitude, respectively, during baseline, 24 h post-treatment, anoxia, and reoxygenation (*** *p* < 0.001, Dunnett’s-Test).

Later, we tested the modulatory effect of SNAP-MSs on hiPSC-CMs by monitoring the real-time electrical and functional activity of cells as depicted in Figure 6A. Our results demonstrate that treating hiPSC-CMs with free SNAP abolished the electrical activity of hiPSC-CMs within 24 h of anoxia treatment leading to cell death. This effect was observed due to the availability of excessive NO from free SNAP, leading to oxidative stress and subsequent cell death (Figure 6B–E). However, when the hiPSC-CMs were treated with SNAP-MSs in low and high doses, there was no change in the electrical activity of cells post-treatment suggesting that the rate of NO release from SNAP-MSs was at non-toxic levels. It is worth mentioning that SNAP-MS-H significantly improved the spike amplitude of treated cells within 24 h of treatment when compared to the control, suggesting that controlled delivery of NO may improve cell functioning. Later, we subjected the cells to anoxic stress by depriving the cells of oxygen. As shown in Figure 6B–E, a gradual decline in the cellular activity was noted in all the groups as evidenced by a low beat rate (Figure 6B,D); nonetheless, SNAP-MS-H demonstrated a significantly improved spike amplitude suggesting that NO could alleviate the cellular dysfunction during anoxic stress. Furthermore, when the cells were subjected to reoxygenation, a gradual decline in the electrical activity was observed in all the groups, which could be due to the ROS produced during the reoxygenation. Our results confirm that controlled NO delivery via SNAP-MSs may improve the electrophysiological parameters of cardiomyocytes and alleviate anoxic stress, but may not rescue cells from ROS damage caused by reoxygenation.

Our current development of sustained NO-releasing MSs demonstrates the potential of a relatively safe and effective delivery modality for such a volatile yet crucial molecule to the human body. However, as much as we initially saw a cardiac use case of the MS, it is important to note that this simplified technique, size, and the properties of the MSs themselves have uses that extend far beyond the cardiovascular system like anti-microbial [42], anti-inflammatory [43], orthopedic regeneration [44], and anti-thrombotic [45]. The uniform shape and slow-releasing nature of the MSs make them an ideal candidate to be used in other areas of the body such as in the case of erectile dysfunction as a prior study has explored [46]. Yet another instance is the potential for these MSs to be used in surgical areas as a prior study has investigated porous microspheres for angiogenesis in bladder reconstruction through the nitric oxide synthase pathway, by loading the microspheres with active adipose mesenchymal stromal cells [47]. In this case, a potentially much more simplified, easily reproducible technique would be to use the slow-releasing NO-MSs for angiogenesis. Other studies have experimented with finding a way to use the vasodilatory effects of NO to address the severe medical condition of pulmonary arterial hypertension through nebulizing NO-releasing MSs deep into the lungs [48]. Our small, uniformly shaped MSs with slow-release capacity and a simplified fabrication technique could potentially be the next ideal candidate to test for therapeutic levels of drug delivery into the highly sensitive areas of the lung. However, beyond simply using the MSs on their own, there is immense potential in coupling them with other therapeutic modalities like nonsteroidal anti-inflammatory drugs (NSAIDs) to minimize the adverse effects and expand the use case of sustained slow-releasing NO [49,50]. Another example is the use of NO-releasing scaffolds for regenerating osteoporotic bone defects [51]. A modular approach can be taken where the scaffold production is simplified, and the actual NO-releasing component can be added on later through electro-spraying. This could allow for using the NO-releasing MS on different types of scaffold depending on what is best suited for the situation, or even employing other MS loaded with different drugs onto a standard or template scaffold. These modular scaffold MS combinations could be used in cases where a more localized impact is desired or possibly when vasodilation or angiogenesis is required in a more cutaneous setting, such as in skin wound healing [52]. Regardless of what specific scenario the slow-releasing NO-MSs are used in, the ease in production, simplicity, and cost-effectiveness of our model demonstrates the immense potential and broadened future possibilities of their use.

## 4. Conclusions

This study focused on developing PCL microspheres loaded with SNAP and assessing the sustained release behavior by employing a modified NO detection method. By adopting the new method, we were able to eliminate the interferences from additives and stabilize and detect NO in real time. We also reported the stability of SNAP when subjected to fabrication parameters, like high-speed homogenization, and the biocompatibility of SNAP-MSs on the HUVEC cell line. We also demonstrated that the effect of controlled delivery of NO to hiPSC-CMs improved the electrophysiological parameters and preserved their function during anoxic stress.

## Data Availability

The data used in this study is available on request.

## Supplementary Materials

The following supporting information can be downloaded at: https://www.mdpi.com/article/10.3390/biomedicines12061363/s1, Figure S1: Detection of NO from SNAP-MS by the conventional method.

## Abbreviations

NO	Nitric Oxide
MS	Microspheres
SNAP	S-Nitroso-N-Acetylpenicillamine
PCL	poly(ε-caprolactone)
GTN	Glyceryl trinitrate
PLGA	poly(lactic-co-glycolic acid)
PVA	Polyvinyl alcohol
SEM	Scanning Electron Microscopy
CuCl_2_	Copper (II) chloride
HUVEC	Human Umbilical Vein Endothelial Cells
DETA NONOate	Diethylenetriamine NONOate
DMSO	Dimethylsulfoxide
RPM	Rotations Per Minute
AUC	Area Under Curve
hiPSC-CMs	human induced Pluoripotent Stem Cell derived Cardiomyocytes
MEA	Multi-Electrode Array
