# Supplementary material for: Fabrication and Optimization of Poly(ε-caprolactone) Microspheres Loaded with S-Nitroso-N-Acetylpenicillamine for Nitric Oxide Delivery
Source: Biomedicines. 2024 Jun 19;12(6):1363. doi: 10.3390/biomedicines12061363 (PMC11201505; doi:10.3390/biomedicines12061363)
Supplement: Supplementary file 1 [file biomedicines-12-01363-s001.zip › biomedicines-2822961-supplementary.pdf]

Supporting Information

Fabrication and Optimization of Poly(ε-caprolactone) Microspheres Loaded with S-Nitroso-N-Acetylpenicillamine for Nitric Oxide Delivery

Syed Baseeruddin Alvi [1], Nooruddin Pracha[1], Mahmoud Shalaan[1], Pankaj Singh Dholaniya[1,4,] Muhamad Mergaye[1], Divya Sridharan[1] and Mahmood Khan[1,2,3*]

[1]Division of Basic and Translational Sciences, Department of Emergency Medicine, The Ohio State University, Columbus, OH 43210, USA.

[2]Department of Physiology and Cell Biology, The Ohio State University, Columbus, OH 43210, USA.

[3]Davis Heart and Lung Research Institute, The Ohio State University, Columbus, OH 43210, USA.

[4]Department of Biotechnology and Bioinformatics, School of Life Sciences, University of Hyderabad, Hyderabad, India.

*Correspondence: Mahmood Khan, Ph.D., FAHA, E-mail; Mahmood.Khan@osumc.edu

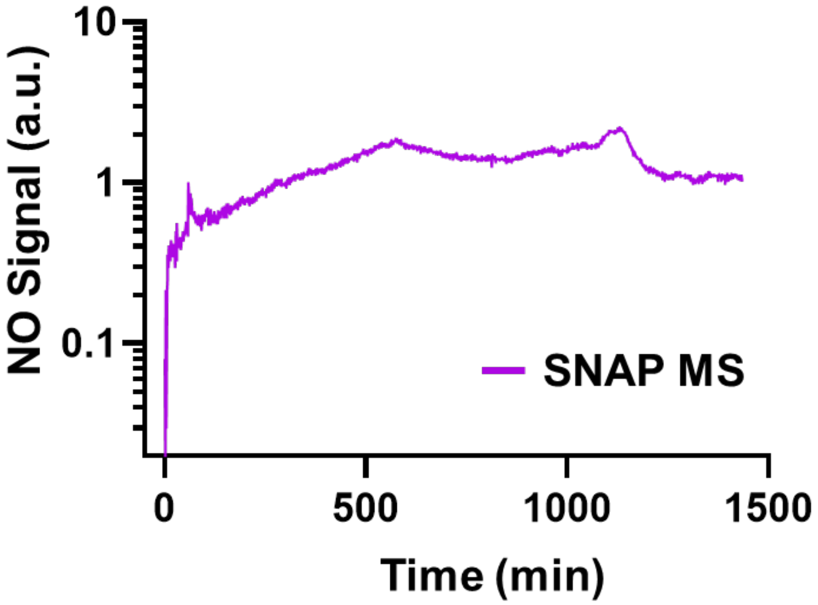

Figure S1: Detection of NO from SNAP-MS by the conventional method. The microfiber-based NO sensor response when immersed in SNAP MS solution.

The NO sensors showed higher noise than signal when immersed in SNAP-MS solution, this response could be due to the interaction of stabilizer or additives from MS with the sensor. Therefore, the modified apparatus was used to eliminate these interactions and acquire the true NO signal.
